# First report of *Escherichia albertii* in Iran: a case study highlighting diagnostic challenges in pediatric gastroenteritis

**DOI:** 10.1128/asmcr.00046-25

**Published:** 2025-08-29

**Authors:** Ali Dadvar, Ali Nemati, Maryam Hafiz, Zahra Khammar, Ute Römling, Mahdi Askari Badouei, Gholamreza Hashemi Tabar

**Affiliations:** 1 Department of Pathobiology, Faculty of Veterinary Medicine, Ferdowsi University of Mashhad 48440, Mashhad, Iran; 2 Department of Microbiology, Tumor and Cell Biology, Biomedicum, Karolinska Institutet 27106, Stockholm, Sweden; 3 Department of Food Science and Technology, School of Nutrition Sciences and Food Technology, Kermanshah University of Medical Sciences 48464, Kermanshah, Iran; Rush University Medical Center, Chicago, Illinois, USA

**Keywords:** *Escherichia albertii*, *Escherichia coli*, Iran, children, PCR, antibiotic

## Abstract

**Background:**

*Escherichia albertii* is a gram-negative, facultative anaerobic bacillus in the order Enterobacterales, family Enterobacteriaceae, increasingly recognized as an emerging enteropathogen. Initially isolated in 1991 from a child with diarrhea in Bangladesh and misclassified as *Hafnia alvei*, it was reclassified in 2003 and is now acknowledged as the second pathogenic species of the *Escherichia* genus, following *Escherichia coli*.

**Case Summary:**

This article reports the first documented case of *E. albertii* in Iran, infecting a 5-year-old girl who presented with profuse watery diarrhea. During a 7-month surveillance study at Akbar Children’s Hospital in Mashhad, stool samples from 231 children with diarrhea were analyzed, with this single case demonstrating infection by *E. albertii*. The strain was identified through pentaplex PCR but exhibited phenotypic traits highly similar to other enteropathogens and showed resistance to antibiotics, such as ampicillin and amoxicillin-clavulanate.

**Conclusion:**

The study highlights the diagnostic challenges associated with the discrimination between *E. coli* and *E. albertii*, which is often misidentified when solely biochemical tests are used. Our findings and literature data suggest that *E. albertii* should be considered a significant cause of pediatric gastroenteritis, akin to *Shigella* and enteroinvasive *E. coli*. These observations emphasize the need for adequate diagnostic protocols, including PCR testing, to accurately identify *E. albertii* and inform appropriate clinical management strategies. Further research is necessary to deepen our understanding of this emerging pathogen and its implications for human health.

## INTRODUCTION

*Escherichia albertii* is a gram-negative, facultative anaerobic bacillus within the Enterobacterales, family Enterobacteriaceae, recognized as an emerging enteropathogen. Initially isolated in 1991 from a 9-month-old child with diarrhea in Bangladesh as *Hafnia alvei* based on biochemical assays (1, 2), subsequent molecular analysis including DNA-DNA hybridization reclassified a group of D-sorbitol- and lactose-negative isolates as a novel species within the *Escherichia* genus in 2003. *E. albertii* has been named in honor of M. John Albert, who described its first isolate (1). This reclassification had demonstrated again the limitation to base taxonomy solely on biochemical tests and highlights the still evolving accuracy in bacterial taxonomy by the application of molecular methodologies such as PCR and whole genome sequence analyses including its significance for accurate identification of isolates in clinical settings.

*E. albertii* is now acknowledged as the second pathogenic species of the *Escherichia* genus, following *Escherichia coli* (3, 4). Its genomic complexity and close evolutionary relationship with both *E. coli* and *Shigella* (which actually belongs to the species *E. coli*) underscore its emerging status as a human and animal enteropathogen (5). With *E. albertii* to be traditionally linked to diarrhea in humans (6), experimental evidence has confirmed its potential to cause gastrointestinal disease, with adherence to epithelial cells identified as a primary virulence trait (7). Infected individuals often present with symptoms typical of gastroenteritis, including watery diarrhea, dehydration, abdominal pain, and, in some cases, fever (8, 9). Besides causing infection in humans, the foodborne pathogen *E. albertii* can also colonize wild and livestock animals, although it was initially not considered a zoonotic pathogen.

The diagnostic challenges posed by *E. albertii* stem from its genetic and phenotypic similarities to other *Escherichia* spp., in particular *E. coli* and *Escherichia fergusonii* (10). However, recently, the presence of a transcriptional activator (EAKF1_ch4033) has been found to be indicative of *E. albertii* (11). The detection of the *eae* gene, also associated with enteropathogenic *Escherichia coli* (EPEC) and enterohemorrhagic *Escherichia coli* (EHEC) strains of *E. coli*, can lead to misidentification of *E. albertii* in routine laboratory tests (12). As a result, *E. albertii* has previously been underreported concomitantly with a lack of awareness regarding its public health implications. This is particularly concerning, given the potential of some *E. albertii* strains to produce Shiga toxin and its association with causing intestinal lesions, which can result in significant morbidity (13–15). The genome of *E. albertii* KF1 is composed of a chromosome of 4,701,875 bp, exhibiting a G + C content of 49.7%, along with four plasmids. The application of multiplex PCR approaches for the identification of various gastrointestinal pathogens, including diarrheagenic *E. coli*, combined with whole genome sequencing for precise identification and typing, has established a more effective and dependable method for the detection and characterization of *E. albertii* (16, 17).

This article reports the first isolation of *E. albertii* from a 5-year-old child in Iran, highlighting its significance as an emerging public health concern. By investigating the prevalence of *E. albertii* in pediatric diarrhea cases, this study seeks to enhance understanding about the occurrence of diarrheal disease caused by *E. albertii* in Iran, the virulence factors involved, and the antimicrobial resistance profiles. The findings, therefore, contribute valuable insights into the epidemiology of this underestimated pathogen, filling a critical gap in knowledge about the pathogenic potential and informing better strategies for prevention and control of related infections in the region.

## CASE PRESENTATION

The Akbar Children’s Hospital, a public facility in Mashhad, Iran, serves patients from across Khorasan and has an active surveillance program for diarrheal pathogens, including *Salmonella*, *Shigella*, *Vibrio* spp., and *Campylobacter*. Diagnosis and management of acute diarrhea in children follow the guidelines set by the Iranian Ministry of Health and the World Health Organization.

The patient, a 5-year-old girl from Mashhad, was previously healthy until May 1, 2022, when she developed profuse watery diarrhea without blood. She was admitted to the Akbar Children’s Hospital on the same day. She had not taken medications prior to disease development and had not been traveling outside Mashhad. Upon admission, tests for *Salmonella* and *Shigella* returned negative. The patient was stable and was discharged after one day in the hospital. However, her stool sample was subsequently sent to the microbiology department for further analysis in the context of a larger study on infectious agents.

### Identification

During a 7-month study period, stool samples from 231 children with diarrhea were collected at the Akbar Children’s Hospital, but none of the samples tested positive for *E. albertii* aside from this case. The strain isolated from the patient’s stool at the hospital produced clear, colorless, circular colonies on MacConkey agar and has subsequently been identified as *E. albertii* using an improved multiplex PCR reaction targeting genes, the presence of which is discriminatory between relevant *E. coli*, relevant pathovars and *Escherichia* spp., namely *E. coli*, *E. albertii*, *E. fergusonii*, *Shigella* spp., and enteroinvasive *E. coli* (EIEC). This PCR approach includes detection of the gene for the cyclic di-GMP regulator CdgR (discriminatory for *E. coli*), for the DNA-binding transcriptional activator of cysteine biosynthesis EAKF1_ch4033 (specific for *E. albertii*), and for the palmitoleoyl-acyl carrier protein-dependent acyltransferase EFER_0790 (specific for *E. fergusonii*). By targeting *ipaH* and *lacY* the PCR assay additionally discriminated between *Shigella* spp. and EIEC (11, 18).

Upon successful amplification of the PCR product specific for *E. albertii*, the gene for the transcriptional activator EAKF1_ch4033 has been sequenced using the BigDye Terminator Cycle Sequencing Kit on an ABI PRISM 3130 Genetic analyzer (Thermo Fisher Scientific) according to the manufacturer’s instruction. EAKF1_ch4033 showed 99% nucleotide sequence identity to the equivalent gene of verified *E. albertii* isolates. The nucleotide sequence has been submitted to GenBank with accession number PV185715.

Moreover, the results of phenotypic and biochemical tests—negative for motility, oxidase, and lactose utilization and positive for catalase, indole, and utilization of mannitol and sorbitol—indicated the isolate being *E. albertii* (Table 1). Some biochemical and even behavioral traits, including sorbitol, lactose, indole, and motility, can, however, vary in *E. albertii*, with some isolates testing positive and others testing negative (19, 20). This variability highlights the diversity within the species. Therefore, these traits should be interpreted with caution when identifying *E. albertii* based on phenotypic tests.

**TABLE 1:** Biochemical properties of the identified *E. albertii* strain

Test	Reaction^*a*^	Test	Reaction
Oxidase	−	Lactose fermentation	−
Catalase	+	Mucate utilization	−
Indole	+	Nitrate reduction	+
Motility	−	Acetate utilization	−
Voges-Proskauer	−	Gelatin hydrolysis	−
Methyl red	+	Eosin methylene blue agar	−
Urease	−	Mannitol salt agar	−
Ortho-nitrophenyl-β-galactoside	+	Xylose fermentation	−
Citrate utilization	−	Deoxyribonuclease	−
Triple sugar iron agar	K/A	Adonitol fermentation	−
Hydrogen sulfide production	−	Mannitol fermentation	+
Lysine decarboxylase	+	D-sorbitol fermentation	+
Bile esculin hydrolysis	−	Salicin fermentation	−
Raffinose fermentation	−		

^*a*^ +, positive reaction; –, negative reaction; K/A, alkaline over acid.

Antimicrobial susceptibility was assessed using the Clinical and Laboratory Standards Institute disk diffusion method, with *E. coli* ATCC 25922 and *Klebsiella pneumoniae* K6 (ATCC 700603) serving as the quality control strain. The *E. albertii* strain was sensitive to trimethoprim-sulfamethoxazole, cefotaxime, meropenem, tetracycline, gentamicin, amikacin, ciprofloxacin, and azithromycin but resistant to ampicillin and amoxicillin-clavulanate. Additionally, we assessed the strain for the presence of 14 resistance-associated genes, including *mcr1-5*, *bla*_TEM_, *bla*_SHV_, *bla*_OXA_, and various *qnr* genes [*qnrA-qnrD*, *qnrS*, and *aac(69)-Ib-cr*] (Table 2). The amplification conditions were similar to previous studies, with minor adjustments to annealing temperatures as needed (21–23). Positive controls were included to confirm the accuracy of the PCR results, using *Klebsiella pneumoniae* (ATCC 700603) and *E. coli* (ATCC 35218), which carry *bla*_TEM_ and *bla*_SHV_, respectively. A number of validated clinical isolates served as the positive controls for *bla*_OXA_, *mcr1-5*, and *qnr* genes. Only the *bla*_TEM_ gene was detected in this strain, while all other resistance genes were absent. Demonstrating consistency between our phenotypic and PCR results, research has shown that the *bla*_TEM_ gene is commonly found in ampicillin-resistant *E. coli* samples from humans (24, 25).

**TABLE 2:** Primers used for the identification of *E. albertii* AD1 and for detecting the presence of virulence and resistance genes in this study

Target gene	Sequence (5′−3′)^*b*^	Primer	Amplicon size (bp)	Reference
Species-specific gene				
EAKF1_ch4033	GTAAATAATGCTGGTCAGACGTTAAGTGTAGAGTATATTGGCAACTTC	Ealb_fw Ealb_rev	393	(18)
Virulence genes				
*eae*	GACCCGGCACAAGCATAAGC CCACCTGCAGCAACAAGAGG	Eae_fw Eae_rev	384	(26)
*stx1*	ATAAATCGCCATTCGTTGACTACAGAACGCCCACTGAGATCATC	Stx1_fw Stx1_rev	180	(26)
*stx2*	GGCACTGTCTGAAACTGCTCC TCGCCAGTTATCTGACATTCTG	Stx2_fw Stx2_rev	255	(26)
*stx2a*	GCGATACTGRGBACTGTGGCC	Stx2a_fw		(27)
	CCGKCAACCTTCACTGTAAATGTG	Stx2a rev1	349	
	GCCACCTTCACTGTGAATGTG	Stx2a rev2	347	
*stx2f*	AGATTGGGCGTCATTCACTGGTTG TACTTTAATGGCCGCCCTGTCTCC	Stx2f fw Stx2f_rev	428	(14)
*ehly*	GCATCATCAAGCGTACGTTCC AATGAGCCAAGCTGGTTAAGCT	ehly_fw ehly_rev	534	(14)
*cnf1,2*	TCGTTATAAAATCAAACAGTC CTTTACAATATTGACATGCTG	CNF_fw CNF_rev	450	(28)
*Est*	GCTAATGTTGGCAATTTTTATTTCTGTA AGGATTACAACAAAGTTCACAGCAGTAA	Sta_fw Sta_rev	190	(28)
*Let*	ATTTACGGCGTTACTATCCTG TTTTGGTCTCGGTCAGATATG	LT_fw LT_rev	280	(28)
*cdtB*	TCGCGGAAGAAGCTATATAC TGGAATATAAATGTCCGGCA	Cdt-B fw Cdt-B_rev	407	This study^*a*^
*iroN*	AATCCGGCAAAGAGACGAACCGCCT GTTCGGGCAACCCCTGCTTTGACTTT	iroN_fw ironN_rev	553	(29)
*ompT*	GTTCGGGCAACCCCTGCTTTGACTTT TCATCCCGGAAGCCTCCCTCACTACTAT	ompT_fw ompT_rev	496	(29)
*Paa*	CGTTGCTGGATGCAGCTAATCTTT CGGCAATATTCATATCACGCCAGA	Paa_fw Paa_rev	561	This study^*a*^
*hlyF*	GGCCACAGTCGTTTAGGGTGCTTACC GGCGGTTTAGGCATTCCGATACTCAG	hlyF_fw hlyF_rev	450	(29)
*iss*	CAGCAACCCGAACCACTTGATG AGCATTGCCAGAGCGGCAGAA	Iss_fw Iss_rev	323	(29)
*iucA*	CGTCATGACTGGACCGCCCTGC GAAGAGGTGGTCGGCAGCCACG	iucA_fw iucA_rev	422	This study^*a*^
*iutA*	GGCTGGACATCATGGGAACTGG CGTCGGGAACGGGTAGAATCG	iutA_fw iutA_rev	302	(29)
Resistance genes				
*bla^TEM^*	CATTTCCGTGTCGCCCTTATTC CGTTCATCCATAGTTGCCTGAC	MultiTSO-T_fw MultiTSO-T_rev	800	(21)
*bla^OXA^*	GGCACCAGATTCAACTTTCAAG GACCCCAAGTTTCCTGTAAGTG	MultiTSO-O_fw MultiTSO-O_rev	564	(21)
*bla^SHV^*	AGCCGCTTGAGCAAATTAAAC ATCCCGCAGATAAATCACCAC	MultiTSO-S_fw MultiTSO-S_rev	713	(21)
*mcr-1*	AGTCCGTTTGTTCTTGTGGC AGATCCTTGGTCTCGGCTTG	mcr1_320bp_fw mcr1_320bp_rev	320	(22)
*mcr-2*	CAAGTGTGTTGGTCGCAGTT TCTAGCCCGACAAGCATACC	mcr2_700bp_fw mcr2_700bp_rev	715	(22)
*mcr-3*	AAATAAAAATTGTTCCGCTTATG AATGGAGATCCCCGTTTTT	mcr3_900bp_fw mcr3_900bp_rev	929	(22)
*mcr-4*	TCACTTTCATCACTGCGTTG TTGGTCCATGACTACCAATG	mcr4_1100bp_fw mcr4_1100bp_rev	1,116	(22)
*mcr-5*	ATGCGGTTGTCTGCATTTATC TCATTGTGGTTGTCCTTTTCTG	MCR5_fw MCR5_rev	1,644	(22)
*qnrA*	CAGCAAGAGGATTTCTCACG AATCCGGCAGCACTATTACTC	qnrA_fw qnrA_rev	630	(23)
*qnrB*	GGCTGTCAGTTCTATGATCG GAGCAACGATGCCTGGTAG	qnrB_fw qnrB_rev	488	(23)
*qnrS*	GCAAGTTCATTGAACAGGGT TCTAAACCGTCGAGTTCGGCG	qnrS_fw qnrS_rev	428	(23)
*qnrD*	CGAGATCAATTTACGGGGAATA AACAAGCTGAAGCGCCTG	qnrD_fw qnrD_rev	581	(23)
*qnrC*	GCAGAATTCAGGGGTGTGAT AACTGCTCCAAAAGCTGCTC	qnrC_fw qnrC_rev	118	(23)
*aac(69)-Ib-cr*	TTGGAAGCGGGGACGGAM ACACGGCTGGACCATA	aac(69)-Ib-cr_fw aac(69)-Ib-cr_rev	260	(23)

^*a*^ The annealing temperatures were 57°C for *cdtB*, 54°C for *Paa*, and 63°C for *iucA*.

^*b*^ R=A,G; B=C,G,T; K=G,T; M=A,C.

Recognizing that certain *E. albertii* strains may encode virulence factors similar to *E. coli*, we conducted single and multiplex PCR analyses to investigate the presence of a fraction of those virulence-associated genes. The genes examined included attachment gene *eae* and *Paa*; toxin genes *stx1*, *stx2*, *stx2a*, *stx2f*, *hlyF*, *elt*, *est*, *cdt*, *iss*, and *ompT*; enterohemolysin *ehlY*; and siderophore genes *iucA*, *iutA*, *and iroN*, all of which contribute to the pathogenicity of *E. coli* (Table 2). *Paa* (porcine attaching and effacing-associated gene coding for the Paa protein) and *cdt* (cytolethal distending toxin gene with its CdtB catalytic subunit) have previously been found in nearly 100% of *E. albertii* isolates (30). Multiple single and multiplex PCR assays were employed, adhering to established protocols (14, 26, 27, 29). *E. coli* O157 strain ATCC 35218 and *E. coli* O157 strain Sakai ATCC BAA-460 were used as positive controls for all these PCR reactions targeting virulence genes. Analysis of the results of the PCR reaction using agarose gel electrophoresis showed the presence of a specific-sized band for the *eae*, *cdtB*, *Paa*, *iucA*, and *iutA* genes (data not shown). The strain, designated *E. albertii* AD1, tested negative for all the other intestinal and extraintestinal virulence genes commonly found in *E. coli*.

## DISCUSSION

*Escherichia albertii* is a gram-negative species increasingly recognized as a cause of human gastroenteritis (31). Historically, biochemical tests have led to misidentifications of *E. albertii* as *E. coli*, *Shigella*, *Yersinia ruckeri*, or *Hafnia alvei* (32). Infections can occur through the consumption of tainted water, vegetables, and meat or contact with contaminated animals (33). This case report marks the first documented occurrence of *E. albertii* in Iran, presenting a rare instance of diarrhea and hospitalization in a 5-year-old girl.

Mild diarrhea was a prominent symptom associated with *E. albertii*-AD1-related gastroenteritis in the patient. More severe pathogenesis may be attributed to virulence factors such as the *stx1* and *stx2* genes, along with the *eae* gene, which are known to interact with intestinal cells, leading to fluid accumulation and symptoms like diarrhea and abdominal pain. These virulence factors can also contribute to damage of the intestinal villi (34).

The biochemical properties of *E. albertii* closely resemble those of *E. coli*, with a few notable differences such as variable motility capacity, depending also on experimental conditions and in the fermentation of distinct sugars. For instance, *E. albertii* has conventionally been considered to be non-motile and unable to ferment certain sugars, including adonitol, xylose, and lactose, within 48 hours (2, 35, 36), while other biochemical traits can be variable. These biochemical and phenotypic similarities and variabilities often lead to its misidentification as *E. coli* and other species in routine biochemical tests (18, 35). In this epidemiological study, *E. albertii* AD1 was accurately identified as *E. albertii* through subsequent informative genomic analysis by multiplex PCR. Of note, phylogenetic grouping with the established multiplex PCR protocol (37) has not been successful (data not shown), providing additional indication for the isolate not belonging to the *E. coli* spp. As a specific molecular approach to identify *E. albertii,* to be generally applicable, the recently established identification of *E. albertii* by matrix-assisted laser desorption/ionization time-of-flight would require the inclusion of a specific database based on verified *E. albertii* isolates.

*E. albertii* AD1 carried a limited number of virulence genes, among them the *eae* adhesion initially thought to be an EPEC and the *Paa* adhesin gene, prevalent in strains of EHEC and ETEC pathovars and previously shown to be present in *E. albertii* (30). The *cdtB* gene was also detected in this strain; the *cdtB* gene in *E. albertii* encodes the active subunit of the cytolethal distending toxin, a tripartite genotoxin being encoded by *cdtA*, *cdtB*, and *cdtC* genes (38). In addition, we detected the *iutA* and *iucA* genes, which are part of the *iucABCD-iutA* operon. This operon encodes the high-affinity aerobactin siderophore system that aids in acquiring iron in iron-limited environments, such as within the host, and is considered an important virulence factor (39).

Although the average incubation period for infection by *E. albertii* is estimated to be 12–24 hours, the specific transmission route remained unclear in this case (32). The prevalence of *E. albertii* gastroenteritis appears to decline significantly with age, dropping from nearly 50% in children aged 0–10 years to 0% in adults over 20 (35). While these findings did not show statistically significant associations, they suggest that younger children are at greater risk of infection compared to adults. Previous studies support this observation, indicating that children under 10 and immunosuppressed individuals may be particularly vulnerable, although outbreaks have been reported in otherwise healthy populations (19).

While the source of infection has not been identified in this presented case, studies suggest that *E. albertii* may have zoonotic potential, with possible animal reservoirs serving as a source of infection. Various animal species, including poultry and livestock, have been implicated as carriers of *E. albertii*, which could be transmitted to humans (40, 41). Other factors such as poor hygiene during food preparation, the consumption of raw or undercooked poultry, and drinking untreated water may elevate the risk for *E. albertii* infection (1). The heightened incidence of clinical disease in younger age groups could reflect either a true association or sampling bias, as enteric infections can have more severe consequences for young children. Additionally, in cases of Shiga toxin-producing *E. coli* infections, children under 5 are more likely to develop hemolytic uremic syndrome (HUS). While not definitively confirmed, it is plausible that the risk of HUS in *E. albertii* infections may also be higher among young children, especially in strains that harbor both the *stx2* and *eae* genes (41).

### Conclusion

This report marks the first documented occurrence of *E. albertii* in Iran, highlighting a rare case of diarrhea and hospitalization in a 5-year-old girl. The strain exhibited resistance to antibiotics such as ampicillin and amoxicillin-clavulanate, underscoring challenges in treating infections caused by this pathogen. The diagnosis of *E. albertii* can be demanding and is often missed, but this case emphasizes the status of *E. albertii* as an emerging pathogen in both humans and animals. The findings show that *E. albertii* can cause diarrhea in children, warranting further investigation about additional virulence factors. Our understanding of its pathobiology, mechanisms of pathogenesis, virulence factors, and drug resistance remains limited even if recently more in-depth analyses have been performed (16, 17). Therefore, suspected *E. albertii* cases need to be interpreted with caution and confirmed through diagnostic PCR tests to ensure accurate diagnosis and appropriate management.

## Data Availability

Data supporting the findings of this study are available within the article; raw data, including agarose gel images, are available from the corresponding author upon reasonable request. The nucleotide sequence of the EAKF1_ch4033 gene from the *E. albertii* AD1 isolate has been submitted to GenBank under accession number PV185715.

## AFTER EPUB

*[This article was published on 29 August 2025 with an error in Table 2. The table was updated in the current version, posted on 12 September 2025.]*

## References

[1] Gomes TAT, Ooka T, Hernandes RT, Yamamoto D, Hayashi T. 2020. Escherichia albertii pathogenesis. EcoSal Plus 9:10-1128. doi:10.1128/ecosalplus.esp-0015-2019 PMC11168576 32588811

[2] Huys G, Cnockaert M, Janda JM, Swings J. 2003. Escherichia albertii sp. nov., a diarrhoeagenic species isolated from stool specimens of Bangladeshi children. Int J Syst Evol Microbiol 53:807–810. doi:10.1099/ijs.0.02475-0 12807204

[3] Gordon DM. 2011. Reservoirs of infection: the epidemiological characteristics of an emerging pathogen, Escherichia albertii. Div Ecol Evol Genet Res Sch Biol Aust Natl Univ Canberra. https://www.agriculture.gov.au/sites/default/files/sitecollectiondocuments/animal-plant/emergency/wildlifeexoticdiseaseprogram/08-09/epidemiological-char-pathogen-albertii.pdf.

[4] Li Q, Wang H, Xu Y, Bai X, Wang J, Zhang Z, Liu X, Miao Y, Zhang L, Li X, Zou N, Yan G, Chen X, Zhang J, Fu S, Fan R, Xu J, Li J, Xiong Y. 2018. Multidrug-resistant Escherichia albertii: co-occurrence of β-lactamase and MCR-1 encoding genes. Front Microbiol 9:258. doi:10.3389/fmicb.2018.00258 29503643 PMC5820351

[5] Retchless AC, Lawrence JG. 2010. Phylogenetic incongruence arising from fragmented speciation in enteric bacteria. Proc Natl Acad Sci U S A 107:11453–11458. doi:10.1073/pnas.1001291107 20534528 PMC2895130

[6] Oaks JL, Besser TE, Walk ST, Gordon DM, Beckmen KB, Burek KA, Haldorson GJ, Bradway DS, Ouellette L, Rurangirwa FR, Davis MA, Dobbin G, Whittam TS. 2010. Escherichia albertii in wild and domestic birds. Emerg Infect Dis 16:638–646. doi:10.3201/eid1604.090695 20350378 PMC3321939

[7] Albert MJ, Faruque SM, Ansaruzzaman M, Islam MM, Haider K, Alam K, Kabir I, Robins-Browne R. 1992. Sharing of virulence-associated properties at the phenotypic and genetic levels between enteropathogenic Escherichia coli and Hafnia alvei. J Med Microbiol 37:310–314. doi:10.1099/00222615-37-5-310 1433251

[8] Albert MJ, Alam K, Islam M, Montanaro J, Rahaman AS, Haider K, Hossain MA, Kibriya AK, Tzipori S. 1991. Hafnia alvei, a probable cause of diarrhea in humans. Infect Immun 59:1507–1513. doi:10.1128/iai.59.4.1507-1513.1991 2004829 PMC257869

[9] Liu Q, Wang H, Zhang S, Yan G, Yang X, Bai X, Deng J, Chen X, Zhang L, Zhang J, Wang B, Zou N, Xiong Y, Zhang Z. 2023. Escherichia albertii isolated from the bloodstream of a patient with liver cirrhosis in China: a case report. Heliyon 9:e22298. doi:10.1016/j.heliyon.2023.e22298 38058622 PMC10695973

[10] Egan M, Ramirez J, Xander C, Upreti C, Bhatt S. 2016. Lambda red-mediated recombineering in the attaching and effacing pathogen Escherichia albertii. Biol Proced Online 18:3. doi:10.1186/s12575-015-0032-8 26843851 PMC4739404

[11] Lindsey RL, Garcia-Toledo L, Fasulo D, Gladney LM, Strockbine N. 2017. Multiplex polymerase chain reaction for identification of Escherichia coli , Escherichia albertii and Escherichia fergusonii. J Microbiol Methods 140:1–4. doi:10.1016/j.mimet.2017.06.005 28599915 PMC5603207

[12] Barmettler K, Biggel M, Treier A, Muchaamba F, Vogler BR, Stephan R. 2022. Occurrence and characteristics of Escherichia albertii in wild birds and poultry flocks in Switzerland. Microorganisms 10:2265. doi:10.3390/microorganisms10112265 36422334 PMC9699108

[13] Nemati A, Askari Badouei M, Hashemi Tabar G, Morabito S, Dadvar A. 2024 Molecular and in silico analyses for detection of Shiga toxin-producing Escherichia coli (STEC) and highly pathogenic enterohemorrhagic Escherichia coli (EHEC) using genetic markers located on plasmid, O Island 57 and O Island 71. BMC Vet Res 20:413. doi:10.1186/s12917-024-04251-0 39272082 PMC11396403

[14] Tomeh R, Nemati A, Hashemi Tabar G, Tozzoli R, Badouei MA. 2024. Antimicrobial resistance, β-lactamase genotypes, and plasmid replicon types of shiga toxin-producing Escherichia coli isolated from different animal hosts. J Appl Microbiol 135:lxae059. doi:10.1093/jambio/lxae059 38467395

[15] Nemati A, Dadvar A, Eppinger M, Karimpour Z, Saberi Kakhki S, Sabeti Moghaddam Sabzevar A, Badouei MA, Gigliucci F, Santos LFD, Nakamura K, Javidi H, Hafiz M. 2025. Shiga toxin-producing Escherichia coli (STEC) in developing countries: a 10-year review with global perspective. Microorganisms 13:1529. doi:10.3390/microorganisms13071529 40732038 PMC12300086

[16] Bengtsson RJ, Baker KS, Cunningham AA, Greig DR, John SK, Macgregor SK, Seilern-Moy K, Spiro S, Chong CC, De Silva PM, Jenkins C, Lawson B. 2023. The genomic epidemiology of Escherichia albertii infecting humans and birds in Great Britain. Nat Commun 14:1707. doi:10.1038/s41467-023-37312-3 36973246 PMC10043262

[17] Fiedoruk K, Daniluk T, Swiecicka I, Murawska E, Sciepuk M, Leszczynska K. 2014. First complete genome sequence of Escherichia albertii strain KF1, a new potential human enteric pathogen. Genome Announc 2:e00004-14. doi:10.1128/genomeA.00004-14 24482506 PMC3907721

[18] Dadvar A, Hashemi Tabar G, Askari Badouei M, Nemati A, Farsiani H. 2023. An improved multiplex-polymerase chain reaction method for simultaneous identification and discrimination of emerging Escherichia and Shigella Species. Int J Enteric Pathog 11:128–133. doi:10.34172/ijep.5621

[19] Muchaamba F, Barmettler K, Treier A, Houf K, Stephan R. 2022. Microbiology and epidemiology of Escherichia albertii - an emerging elusive foodborne pathogen. Microorganisms 10:875. doi:10.3390/microorganisms10050875 35630320 PMC9145129

[20] Carter MQ, Carychao D, Lindsey RL. 2024. Conditional expression of flagellar motility, curli fimbriae, and biofilms in Shiga toxin- producing Escherichia albertii Front Microbiol 15:1456637. doi:10.3389/fmicb.2024.1456637 39318426 PMC11420993

[21] Dallenne C, Da Costa A, Decré D, Favier C, Arlet G. 2010. Development of a set of multiplex PCR assays for the detection of genes encoding important beta-lactamases in Enterobacteriaceae. J Antimicrob Chemother 65:490–495. doi:10.1093/jac/dkp498 20071363

[22] Rebelo AR, Bortolaia V, Kjeldgaard JS, Pedersen SK, Leekitcharoenphon P, Hansen IM, Guerra B, Malorny B, Borowiak M, Hammerl JA, Battisti A, Franco A, Alba P, Perrin-Guyomard A, Granier SA, De Frutos Escobar C, Malhotra-Kumar S, Villa L, Carattoli A, Hendriksen RS. 2018. Multiplex PCR for detection of plasmid-mediated colistin resistance determinants, mcr-1, mcr-2, mcr-3, mcr-4 and mcr-5 for surveillance purposes. Euro Surveill 23:17–672. doi:10.2807/1560-7917.ES.2018.23.6.17-00672 PMC5824125 29439754

[23] Ciesielczuk H, Hornsey M, Choi V, Woodford N, Wareham DW. 2013. Development and evaluation of a multiplex PCR for eight plasmid-mediated quinolone-resistance determinants. J Med Microbiol 62:1823–1827. doi:10.1099/jmm.0.064428-0 24000223

[24] Gundran RS, Cardenio PA, Villanueva MA, Sison FB, Benigno CC, Kreausukon K, Pichpol D, Punyapornwithaya V. 2019. Prevalence and distribution of blaCTX-M, blaSHV, blaTEM genes in extended- spectrum β- lactamase- producing E. coli isolates from broiler farms in the Philippines. BMC Vet Res 15:227. doi:10.1186/s12917-019-1975-9 31277658 PMC6612079

[25] Hemeg HA. 2018. Molecular characterization of antibiotic resistant Escherichia coli isolates recovered from food samples and outpatient Clinics, KSA. Saudi J Biol Sci 25:928–931. doi:10.1016/j.sjbs.2018.01.016 30108443 PMC6087806

[26] Paton AW, Paton JC. 1998. Detection and characterization of shiga toxigenic Escherichia coli by using multiplex PCR assays for stx1, stx2, eaeA, enterohemorrhagic E. coli hlyA, rfbO111, and rfbO157. J Clin Microbiol 36:598–602. doi:10.1128/JCM.36.2.598-602.1998 9466788 PMC104589

[27] Scheutz F, Teel LD, Beutin L, Piérard D, Buvens G, Karch H, Mellmann A, Caprioli A, Tozzoli R, Morabito S, Strockbine NA, Melton-Celsa AR, Sanchez M, Persson S, O’Brien AD. 2012. Multicenter evaluation of a sequence-based protocol for subtyping shiga toxins and standardizing Stx nomenclature. J Clin Microbiol 50:2951–2963. doi:10.1128/JCM.00860-12 22760050 PMC3421821

[28] Askari Badouei M, Morabito S, Najafifar A, Mazandarani E. 2016. Molecular characterization of enterohemorrhagic Escherichia coli hemolysin gene (EHEC-hlyA)-harboring isolates from cattle reveals a diverse origin and hybrid diarrheagenic strains. Infect Genet Evol 39:342–348. doi:10.1016/j.meegid.2016.02.002 26855346

[29] Johnson TJ, Wannemuehler Y, Doetkott C, Johnson SJ, Rosenberger SC, Nolan LK. 2008. Identification of minimal predictors of avian pathogenic Escherichia coli virulence for use as a rapid diagnostic tool. J Clin Microbiol 46:3987–3996. doi:10.1128/JCM.00816-08 18842938 PMC2593276

[30] Leszczyńska K, Święcicka I, Daniluk T, Lebensztejn D, Chmielewska-Deptuła S, Leszczyńska D, Gawor J, Kliber M. 2023. Escherichia albertii as a potential enteropathogen in the light of epidemiological and genomic studies. Genes (Basel) 14:1384. doi:10.3390/genes14071384 37510288 PMC10379040

[31] Huang S, Liu Q, Fang Y, Yu H, Yang X, Hu J, Wang Y, Tian R, Gao Y, Ni Z, Xiong Y. 2024. An outbreak associated with Escherichia albertii in a junior high school, China. Epidemiol Infect 152:e117. doi:10.1017/S0950268824001341 39363601 PMC11450498

[32] Ooka T, Seto K, Kawano K, Kobayashi H, Etoh Y, Ichihara S, Kaneko A, Isobe J, Yamaguchi K, Horikawa K, Gomes TAT, Linden A, Bardiau M, Mainil JG, Beutin L, Ogura Y, Hayashi T. 2012. Clinical significance of Escherichia albertii. Emerg Infect Dis 18:488–492. doi:10.3201/eid1803.111401 22377117 PMC3309589

[33] Konno T, Suzuki S, Takahashi S, Kashio H, Ito Y, Kumagai Y. 2021. Isolation and characterization of Escherichia albertii from retail meats and vegetables in akita prefecture, Japan. Jpn J Food Microbiol 38:144–152. doi:10.5803/jsfm.38.144

[34] Sulaiman MA, Aminu M, Ella EE, Abdullahi IO. 2021. Prevalence and risks factors of the novel Escherichia albertii among gastroenteritis patients in Kano State, Nigeria. J Med Trop 23:39–45. doi:10.4103/jomt.jomt_34_20

[35] Nimri LF. 2013. Escherichia albertii, a newly emerging enteric pathogen with poorly defined properties. Diagn Microbiol Infect Dis 77:91–95. doi:10.1016/j.diagmicrobio.2013.06.028 23938305

[36] Abbott SL, O’Connor J, Robin T, Zimmer BL, Janda JM. 2003. Biochemical properties of a newly described Escherichia species, Escherichia albertii. J Clin Microbiol 41:4852–4854. doi:10.1128/JCM.41.10.4852-4854.2003 14532241 PMC254372

[37] Clermont O, Bonacorsi S, Bingen E. 2000. Rapid and simple determination of the Escherichia coli phylogenetic group. Appl Environ Microbiol 66:4555–4558. doi:10.1128/AEM.66.10.4555-4558.2000 11010916 PMC92342

[38] Hyma KE, Lacher DW, Nelson AM, Bumbaugh AC, Janda JM, Strockbine NA, Young VB, Whittam TS. 2005. Evolutionary genetics of a new pathogenic Escherichia species: Escherichia albertii and related Shigella boydii strains. J Bacteriol 187:619–628. doi:10.1128/JB.187.2.619-628.2005 15629933 PMC543563

[39] Russo TA, Olson R, Macdonald U, Metzger D, Maltese LM, Drake EJ, Gulick AM. 2014. Aerobactin mediates virulence and accounts for increased siderophore production under iron-limiting conditions by hypervirulent (hypermucoviscous) Klebsiella pneumoniae. Infect Immun 82:2356–2367. doi:10.1128/IAI.01667-13 24664504 PMC4019165

[40] Masuda K, Ooka T, Akita H, Hiratsuka T, Takao S, Fukada M, Inoue K, Honda M, Toda J, Sugitani W, Narimatsu H, Ishioka T, Hirai S, Sekizuka T, Kuroda M, Morita Y, Hayashi T, Kimura H, Oishi K, Ohnishi M, Fujimoto S, Murakami K. 2020. Epidemiological aspects of Escherichia albertii outbreaks in Japan and genetic characteristics of the causative pathogen. Foodborne Pathog Dis 17:144–150. doi:10.1089/fpd.2019.2654 31603704

[41] Torkan A, Badouei MA. 2024. The evolving menace: emerging *Escherichia* species and their implications for animals and public health, p 1–25. In Epizootics - outbreaks of animal disease. InTechOpen.

